# Psychotherapy During COVID-19: How the Clinical Practice of Italian Psychotherapists Changed During the Pandemic

**DOI:** 10.3389/fpsyg.2020.591170

**Published:** 2020-10-21

**Authors:** Tommaso Boldrini, Arianna Schiano Lomoriello, Franco Del Corno, Vittorio Lingiardi, Silvia Salcuni

**Affiliations:** ^1^Department of Developmental Psychology and Socialization, University of Padova, Padova, Italy; ^2^Department of Cognitive System, Denmark Technical University (DTU), Copenhagen, Denmark; ^3^Association for Research in Clinical Psychology (ARP), Milan, Italy; ^4^Department of Dynamic and Clinic Psychology, Faculty of Medicine and Psychology, Sapienza University of Rome, Rome, Italy

**Keywords:** telepsychotherapy, COVID-19, public health, remote psychotherapy, psychotherapy

## Abstract

**Aims:** Italy was one of the first countries to be significantly affected by the coronavirus disease 2019 (COVID-19) pandemic, determining a unique scenario for Italian psychotherapists to consider changing the modality in which they deliver treatment. The present study aimed at studying which factors related to psychotherapists and their clinical practice had a major role in predicting two main outcomes: (1) the rate of interrupted treatments during lockdown and (2) psychotherapists’ satisfaction with the telepsychotherapy modality.

**Methods:** An online survey was administered to licensed psychotherapists (*n* = 306), who worked mainly as private practitioners, between April 5 and May 10, 2020 (i.e., the peak of the pandemic in Italy).

**Results:** Psychotherapists reported that 42.1% (SD = 28.9) of their treatments had been interrupted, suggesting that Italy faced an important undersupply of psychotherapy during the lockdown. Using the Akaike information criterion (AIC) model selection, we identified three predictors of the rate of interrupted treatments: (1) psychotherapists’ lack of experience with telepsychotherapy prior to the lockdown, (2) their theoretical orientation (with cognitive behavioral psychotherapists reporting a higher rate of interrupted treatments), and (3) patients’ lack of privacy at home, as reported to the psychotherapists. Furthermore, we found four predictors of psychotherapists’ satisfaction with the telepsychotherapy modality: (1) the rate of interrupted treatments, (2) psychotherapists’ previous experience with telepsychotherapy, (3) their beliefs about the compatibility of telepsychotherapy with their theoretical orientation, and (4) their use of a video-conferencing modality, rather than telephone.

**Conclusion:** The following recommendations can help policy makers, professional associations, and practitioners in promoting the continuity of psychotherapy treatments during the COVID-19 outbreak and in future emergencies: (i) disseminating training programs for practitioners on telepsychotherapy, (ii) supporting patients to pragmatically access a private space at home, (iii) encouraging practitioners to use video-conferencing (instead of telephone) to deliver remote therapy, and (iv) increasing the acceptance of telepsychotherapy among both clinicians and the general public.

## Introduction

Italy was one of the first countries to be severely affected by the coronavirus disease 2019 (COVID-19). Beginning on February 23, 2020, the Italian government took strong actions to restrict residents’ freedom, aimed at reducing the contagion. The most severe of these restrictions was the imposition of a nationwide lockdown in early March. This lockdown caused unprecedented changes in daily personal and professional activities, forcing Italian residents to avoid unnecessary face-to-face interactions and social gatherings, as well as limiting their movement to the strictly necessary.

Along with other healthcare treatments, psychotherapy was not subject to the full government restrictions, with the exception of general precautions (i.e., as outlined in the Italian Ministerial Decree of March 8, 2020). However, while it remained possible to maintain in-person psychotherapy sessions, doing so was practically challenged in private clinics and public health systems, considering that face-to-face meetings could increase the risk of infection for both therapists and patients; thus, the National Council of Psychologists CNOP) explicitly invited psychologists and psychotherapists, as far as possible, to provide their professional services *via* digital devices to guarantee the continuation of previously active therapeutic treatments and to ensure the mental health support for diseases linked to pandemic and quarantine. Guidance and regulation for telepsychology in Italy was provided in a document on recommendations for telepsychology [National Council of Psychologists (CNOP), 2017], which did not forbid any online psychological practices, and provided specific guidelines regarding deontological norms, informed consent, privacy and correct identification of users, and emergency situations management (i.e., recommending therapist to obtain emergency numbers and contact details of places offering support that are close by the place where a patient logs in or telephones). The natural consequence of this extraordinary situation was that a primary element of psychotherapy – the setting – was subject to renewed reflection. Specifically, the crossroad at which psychotherapists found themselves was defined by a choice between using telepsychotherapy – which offered the possibility of continuing therapy – or temporarily interrupting treatment.

At this historical time, the continuity of care for psychological treatment is pivotal. A recent study by Brooks et al. (2020) documented an increase in mental health disorders due to the COVID-19 pandemic, including self-reported symptoms of anxiety and depression (16–28%), and stress (8%), frequently in association with a sleep disorder (Rajkumar, 2020).^1^ Evidence suggests that telepsychotherapy could represent a safe and efficacious alternative to physical treatment during the pandemic (Swartz, 2020; Wind et al., 2020). Poletti et al. (2020) reviewed the results of 18 empirical studies in which psychotherapy was provided *via* synchronous web technology. Interestingly, the authors reported that telepsychotherapy was substantially equivalent to face-to-face psychotherapy in its efficacy for treating common mental health disorders (Poletti et al., 2020). In particular, research has found telepsychotherapy to be effective in treating anxiety (Catarino et al., 2018), depressive (Egede et al., 2015; Catarino et al., 2018), and posttraumatic symptoms (Wierwille et al., 2016). Of note, patients who attend telepsychotherapy treatments report similar perceived quality of life, satisfaction, and treatment credibility as those enrolled in face-to-face psychotherapy (Egede et al., 2015).

Conversely, despite the evidence for its effectiveness, negative attitudes about telepsychotherapy are prevalent (see also Varker et al., 2018). Survey studies have reported that approximately half of all respondent psychotherapists perceive telepsychotherapy as less effective than face-to-face psychotherapy (Gordon et al., 2015, 2016; Schulze et al., 2018). Indeed, there are ethical arguments against the seamless implementation of online therapy, including (1) privacy, confidentiality, and security issues, (2) therapist competence and need for special training, (3) communication issues specific to technology, (4) research gaps, and (5) emergency issues (Stoll et al., 2020).

Moreover, patients have been found to express a low willingness to use telepsychotherapy (Apolinário-Hagen et al., 2017; Hantsoo et al., 2017), especially when they have already experienced face-to-face psychotherapy (Hantsoo et al., 2017). General skepticism toward telepsychotherapy is also present and is particularly strong among practitioners. In a sample of 1,791 US psychotherapists, nearly 80% reported that they did not use telepsychotherapy within their own practice (Pierce et al., 2019). Overall, learning curves in the adoption of new e-mental health technologies by both patients and psychologists have progressed far more slowly than initially expected, thus tallying with the estimate that it takes, on average, 16 years for a healthcare innovation to be implemented (Rogers et al., 2017). However, this prevision has been dramatically disproved by the COVID-19 lockdown, which has led to significant and swift changes in clinical practice. This, in turn, has given rise to a unique opportunity to study the consequences of a sudden, large-scale, massive setting transition towards telepsychotherapy.

As Italy was one of the first countries to experience forced changes in clinical activities due to the COVID-19 pandemic, the aim of the present investigation was to provide a picture of the scenario and to delineate which factors played a pivotal role in promoting better telepsychotherapy interventions at this time. In doing so, the investigation sought to generate knowledge to guide other countries struggling with the pandemic. For this purpose, we focused on two outcomes: (1) the rate of interrupted treatments (i.e., failure in the implementation of telepsychotherapy treatments) and (2) psychotherapists’ satisfaction with the telepsychotherapy modality. In particular, we collected information related to the psychotherapists (e.g., sociodemographic characteristics, theoretical orientation, and treatment modality), their clinical practice (e.g., their selected modality for delivering remote psychotherapy sessions, previous experience with telepsychotherapy), and their general beliefs about telepsychotherapy (e.g., their perception of the compatibility of their theoretical orientation to the online modality), as these factors were thought to play a role in determining the selected outcomes.

## Materials and Methods

### Study Design

An online survey designed in Qualtrics was administered to licensed psychotherapists in Italy, using snowball sampling techniques. Data were collected from April 5 to May 10, 2020 – during the peak of the pandemic in Italy, approximately 5 weeks from the beginning of the lockdown and just before the second phase of restrictions easement (e.g., to allow access to church services, weddings, salon services, and short-term hospitality without boarding).

Participation in the research was voluntary, and no incentives were provided. All participants provided informed consent by agreeing to the data protection declaration prior to starting the survey. The principles outlined in the Declaration of Helsinki were followed, ensuring anonymous participation through the administration of the informed consent format of the ethics committee of the University of Padua (GDPR EU 2016, pd. 196/03).

### Description of Study Participants

**Figure 1:**
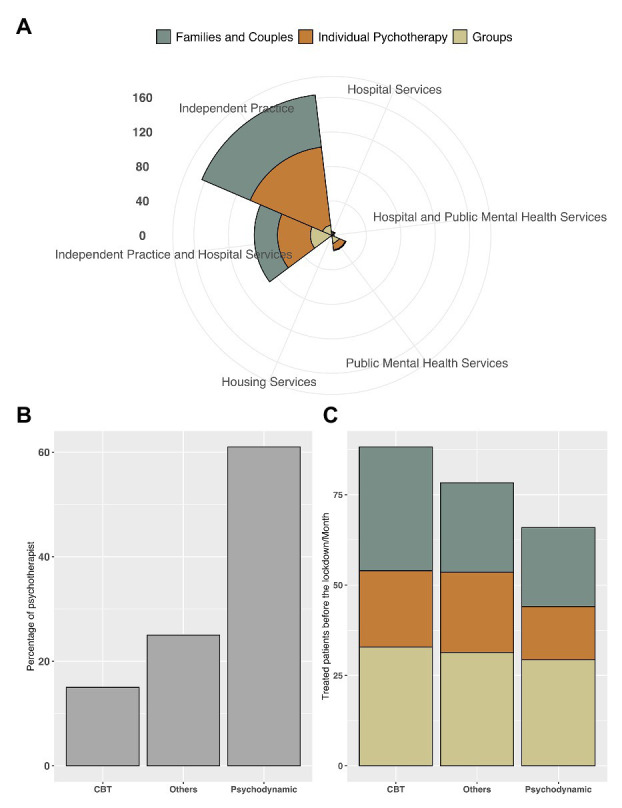
The respective plots depict **(A)** the number of patients in treatment during the month prior to lockdown as a function of psychotherapists’ work settings (i.e., independent practices, hospitals, public mental health, and housing services) and patient orientation (i.e., family and couples, individual, and group); **(B)** psychotherapists’ theoretical orientations [i.e., psychodynamic, cognitive behavioral therapy (CBT), and “other”]; and **(C)** the proportion of patients in treatment in the month prior to lockdown, as a function of clinicians’ theoretical orientation (i.e., psychodynamic, CBT, and “other”) and patient orientation (i.e., family and couples, individual, and group).

A sample of 308 psychotherapists [84% female; mean age = 45.1 (SD = 10.2)] completed the survey. The geographical provenance of the respondents was pretty homogeneous (Northern Italy = 37%, Central Italy = 35%, Southern Italy = 28%). Participants had been registered psychotherapists in Italy for mean = 12.9 (SD = 8.5) years, and they typically (i.e., before the COVID-19 lockdown) treated an average of 21.8 patients (SD = 16.3) per month. Their psychotherapeutic orientations were as follows: psychodynamic (60.8%), cognitive behavioral (16.1%), systemic (8.6%), humanistic (11.7%), and integrated (2.27%). Individual psychotherapy was the preferred treatment modality of 49.1% of the clinicians; 32% saw mostly families and couples; and the rest (18%) specialized in group therapy. The enrolled psychotherapists performed their work mainly as private practitioners (58.4%), with most of the rest (32%) working in hospitals or mental health services in addition to private practice (see also Figure 1). Finally, the majority of the enrolled psychotherapists, under ordinary circumstances (i.e., before the COVID-19 lockdown), received clinical supervision: 38% received one supervision session per month, 36.3% received two to four sessions per month, and 5.34% received more than four sessions each month. The remaining psychotherapists (20.3%) received no supervisions. Information about the therapists’ clinical practice is summarized in Figure 1.

### Measures

The survey comprised 45 items in total, and it took respondents approximately 8 min to complete. Given the aim of the present study, we analyzed only a portion of all the items of the survey. In addition to collecting sociodemographic characteristics and information about the psychotherapists’ working practices (as reported above), the survey also asked respondents to report the proportion of their interrupted treatments since the COVID-19 lockdown, as well as the relative proportions of their patients whom they currently treated face-to-face, *via* telephone, and *via* video-conferencing. Respondents were also asked to rate their personal beliefs about telepsychotherapy, in terms of its compatibility with their therapeutic orientation, and their personal satisfaction with it. Additional items evaluated respondents’ previous experience with video psychotherapy and if their patients reported a lack of access to private space at home.

### Statistical Analysis

In the following analyses, we adopted a model selection strategy based on the Akaike information criterion (AIC; Wagenmakers and Farrell, 2004). The AIC (Akaike, 1973) is a powerful metric derived from information theory that identifies the relative quality of each model within a set of candidate models (i.e., the lower the AIC, the higher the model quality, after controlling for model complexity).

Each full model was compared with simpler versions by removing predictors until an intercept-only model was reached. After identifying the best model (with the lowest AIC), we regressed participants’ responses on the same set of regressors. Significant effects were explored with *post hoc* pairwise contrast using the Wald test, corrected for multiple comparisons using the false discovery rate (Benjamini and Hochberg, 1995).

The analyses were performed using the software R (2.13) with the lm function from the car package (Fox and Weisberg, 2019).

## Results

### Changes in the Provision of Psychotherapy During the COVID-19 Lockdown

With respect to psychotherapists’ changes in clinical practice during the COVID-19 lockdown, respondents reported that 42.1% (SD = 28.9) of their psychotherapy treatments were interrupted during the lockdown.^2^ The remainder of their treatments was primarily delivered *via* online video [63.7% (SD = 38.3)] or telephone [29.1% (SD = 25.3)]. Only 7.2% (SD = 15.1) of their treatments were delivered face-to-face, while taking precautionary measures (e.g., wearing masks and gloves).

### Predictors of a Higher Rate of Interrupted Treatments

The rate of interrupted treatments was estimated *via* a linear model. Data were fit to one model, which included respondents’ therapeutic modality (individual vs. couples and families vs. groups), theoretical orientation [psychodynamic vs. cognitive behavioral therapy (CBT) vs. “other”], clinical experience (in years), previous experience with telepsychotherapy (frequent vs. rare vs. none), beliefs about the compatibility of telepsychotherapy with their own theoretical orientation (yes vs. no), and frequency of supervisions received before the outbreak (none vs. once vs. one to four vs. more than four per month), as well as patient’s reported lack of privacy at home (yes vs. no) as predictive variables, as well as the interactions between these variables.^3^

Model comparisons showed that the best model for explaining the data observed for the rate of interrupted treatments included clinicians’ previous experience with telepsychotherapy, clinicians’ theoretical orientation, and patient’s reported lack of privacy at home as predictive variables (AIC = 2,656.3, logL = −1,320.12, ΔAIC = 35.02).^4^

**Figure 2:**
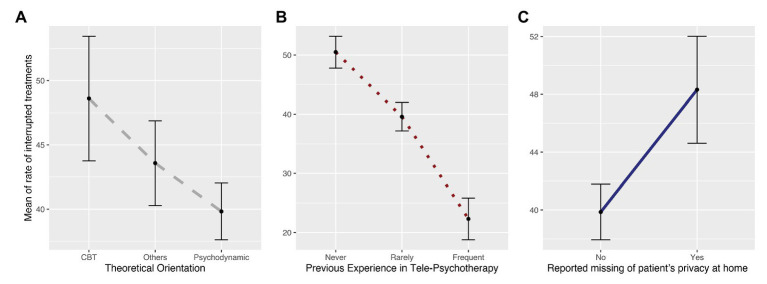
The plots depict the parameters selected as the best predictors of the rate of interrupted treatments. In particular, they represent differences between **(A)** theoretical orientations (i.e., psychodynamic, CBT, and “other”); **(B)** psychotherapists’ use of telepsychotherapy prior to the lockdown (i.e., none, rare, and frequent); and **(C)** patients’ lack of privacy at home, as reported to psychotherapists (i.e., yes, no).

We regressed participants’ responses to these sets of regressors and found a significant difference predicted by clinicians’ previous experience with telepsychotherapy (*b* = −11.53, SE = 3.47, *p* = 0.001), suggesting that the rate of interrupted treatments was significantly lower when psychotherapists reported having frequently used telepsychotherapy prior to the COVID-19 outbreak (*μ* = 22.3; SD = 21.75); the opposite was true when psychotherapists reported having never used this modality (*μ* = 50.50; SD = 29.94). Notably, a significant difference was also present for psychotherapists who reported having used this modality rarely (*μ* = 39.54; SD = 26.40), compared to those who reported either frequent or no previous experience with telepsychotherapy. Furthermore, the model showed a significant difference depending on respondents’ theoretical orientation (*b* = −12.04, SE = 4.75, *p* = 0.01), whereby those with a psychodynamic approach reported a lower rate of interrupted treatments (*μ* = 39.82; SD = 28.86), compared to those practicing CBT (*μ* = 48.6; SD = 31.11). However, this difference was not significant with those characterized as having an “other” clinical orientation. Finally, we found a significant effect of patients’ reported lack of privacy at home (*b* = 10.37, SE = 3.77, *p* = 0.006), suggesting that psychotherapists with patients lacking private space at home (*μ* = 48.31; SD = 31.45) experienced a significantly higher rate of interrupted treatments compared to those who did not report the same issue (*μ* = 39.86; SD = 27.74; Figure 2 and Supplementary Table S1).

### Predictors of Therapists’ Satisfaction With Telepsychotherapy

Participants’ satisfaction was estimated *via* a generalized linear effect binomial model because the outcome variable (yes vs. no) was dichotomous. Data were fit in a model that included respondents’ theoretical orientation (psychodynamic vs. CBT vs. other), clinical experience (in years), previous experience with telepsychotherapy (frequent vs. rare vs. none), beliefs about the compatibility of telepsychotherapy with their own theoretical orientation (yes vs. no), rate of interrupted treatments, dropped clinical supervisions (none vs. half vs. more than the half vs. all), use of the telephone, use of video-conferencing, and therapeutic modality (individual vs. couples and families vs. groups) as predictive variables, as well as the interactions between these variables.^5^

Model comparison showed that the model that best explained the data observed for perceived satisfaction included the rate of interrupted treatments, previous experience with telepsychotherapy, theoretical compatibility, and use of video-conferencing as predictive variables (AIC = 305.5, logL = −146.618, ΔAIC = 2,386.01).

**Figure 3:**
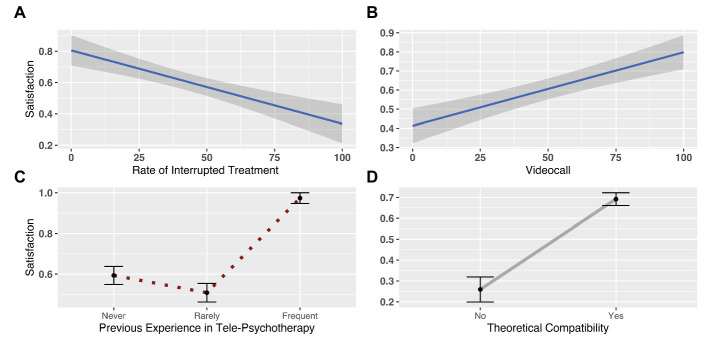
The plots depict the parameters selected as the best predictors of differences in psychotherapists’ satisfaction with telepsychotherapy. In particular, the respective plots represent the variation in perceived satisfaction according to **(A)** the rate of interrupted treatments; **(B)** the use of video-conferencing to deliver sessions; **(C)** psychotherapists’ use of telepsychotherapy prior to the lockdown (i.e., none, rare, and frequent); and **(D)** psychotherapists’ beliefs about the compatibility of telepsychotherapy with their theoretical orientation (i.e., yes, no).

We regressed participants’ responses to these sets of regressors and found a significant difference in satisfaction determined by psychotherapists’ rate of interrupted treatments (*b* = −0.01, SE = 0.005, *p* = 0.02), whereby the more satisfaction they declared, the less dropout they reported. We also found a significant effect of previous experience with telepsychotherapy (*b* = 2.43, SE = 1.05, *p* = 0.02), indicating that psychotherapists who reported having frequently used telepsychotherapy prior to the COVID-19 lockdown had significantly higher satisfaction (*μ* = 0.97; SD = 0.16) than those who reported having never used this modality (*μ* = 59.35; SD = 29.94). Notably, a significant difference was also found for psychotherapists who reported having rarely used this modality (*μ* = 0.51; SD = 0.50), compared to those who had either frequent or no previous experience with telepsychotherapy. Furthermore, the model showed a significant effect of theoretical compatibility (*b* = 1.62, SE = 0.38, *p* < 0.001), suggesting that psychotherapists who perceived their theoretical orientation as compatible with the telepsychotherapy modality (*μ* = 0.69; SD = 0.46) were more satisfied than those who perceived their orientation as incompatible (*μ* = 0.26; SD = 0.44). The model also revealed a significant effect of the number of video-conference calls (*b* = 0.01, SE = 0.004, *p* = 0.01), showing that the more psychotherapists provided sessions *via* video-conferencing, the more satisfaction they reported (Figure 3; Supplementary Table S2).

## Discussion

The present study aimed at identifying the most significant factors in delivering psychotherapy during the COVID-19 lockdown in Italy. In this vein, we sought to both describe the situation for psychotherapy during the peak of the pandemic in Italy and provide guidance for countries still facing (or likely to face) a similar situation as that experienced in Italy.

The first outcome of the present study considered the rate of interrupted treatments (as reported by psychotherapists), suggesting the degree of failure in implementing telepsychotherapy. Psychotherapists reported that 42.1% of their treatments had been interrupted, suggesting that, during the COVID-19 lockdown, there was an important undersupply of psychotherapy. These data are even more surprising, because the majority of participants worked exclusively (58.4%) or mainly (32%) as private practitioner, so they could potentially quickly rethink their clinical practice without having to conform to the slower reorganization that impacted on public mental health services and hospitals. A similar reduction in psychotherapy was observed in Austria, where a decline in face-to-face sessions was compensated by a reported increase in telepsychotherapy in the early weeks of the COVID-19 lockdown – even though the increase was not sufficient to cover the full proportion of interrupted treatments (Probst et al., 2020). On the contrary, a survey study conducted in the Czech Republic, Germany, and Slovakia did not observe psychotherapy dropout during the pandemic (Humer et al., 2020). Notably, the present study was conducted during the peak of the pandemic, after the Italian government imposed strong limitations on personal movement; in contrast, no curfews existed in the aforementioned survey study at the time of data collection (Humer et al., 2020). This mismatch in the study conditions substantially limits our ability to compare findings.

The present analyses focused on identifying the predictive factors of treatment interruption, emphasizing that both therapists and patients play a role in this outcome. In particular, the model of best fit suggested that psychotherapists’ lack of experience with telepsychotherapy prior to the lockdown was an essential factor in predicting the rate of interrupted treatments. It is reasonable to assume that a lack of experience with telepsychotherapy may have threatened clinicians’ professional self-confidence (Poletti et al., 2020). Further, a lack of familiarity with using technology to provide video-conferencing psychotherapy (present in 43.8% of our sample) could have represented a barrier to providing remote treatment, as suggested by previous research (Rössler et al., 2011; Cipolletta et al., 2017; Etzelmueller et al., 2018). This finding is corroborated by evidence that therapists who have received specific training in delivering telepsychotherapy are more likely to adopt this treatment modality (Pierce et al., 2020). Given that COVID-19 may impact nearly everyone in the world, the need for psychological support is fundamental (Duan and Zhu, 2020). Thus, the dissemination of training programs on telepsychotherapy and video-conferencing technology by professional associations may be crucial for countries affected by the pandemic, in order to prevent an undersupply of psychotherapy treatment.

A secondary relevant aspect shown in our analyses related to patients. Although the information we obtained on patients’ conditions was derived from psychotherapists, the data suggested that patients’ reported lack of private space at home presented a significant barrier to the implementation of telepsychotherapy. According to this finding, the continuation of therapeutic work may require therapists to pragmatically discuss with their patients the incidental difficulties in achieving an intimate, reassuring, and safeguarded setting in which to participate in telepsychotherapy sessions.

Moreover, we found a significant effect of theoretical orientation on the rate of interrupted treatments, with psychodynamic therapists reporting a lower rate than CBT therapists. This result is unexpected, since previous studies have found CBT clinicians to be more accepting of telehealth interventions than psychodynamic therapists (e.g., Perle et al., 2012), and similar evidence is deducible from the higher number of studies on CBT delivered remotely (e.g., Egede et al., 2015; Zerwas et al., 2017; Catarino et al., 2018; Etzelmueller et al., 2018).

The second focus of the present investigation was psychotherapists’ satisfaction with telepsychotherapy. As expected, the rate of interrupted treatments represented a negative predictor, as it implied a withdrawal of therapists’ professional duties and consequent financial damage. Importantly, among the two different modalities for delivering remote sessions (i.e., telephone vs. video-conferencing), only the video-conferencing modality predicted therapists’ satisfaction, suggesting that – although telephonic communication may provide a fast and easy method of providing remote care – whenever possible, therapists should choose video-conferencing technology over the telephone. Indeed, previous studies have reported the efficacy of this modality, explaining that it enables psychotherapists and patients in separate locations to see each other and interact in real time (i.e., “synchronously”; see Fletcher et al., 2018; Norwood et al., 2018, for reviews). This finding is also supported by experimental studies showing that the perceived distance between two interacting individuals modulates the empathic reaction between them (Schiano Lomoriello et al., 2018), which is a key ingredient of all psychotherapeutic interventions.^6^

Finally, therapists’ attitudes and beliefs about telepsychotherapy played a significant role in qualitatively shaping their experiences of online sessions. In particular, we found that therapists who considered the online modality as incompatible with their theoretical orientation reported less satisfaction. Overall, there are no valid reasons to believe that a specific therapeutic orientation is more or less suitable for telepsychotherapy (Varker et al., 2018; Poletti et al., 2020). In fact, preliminary research has pointed to the efficacy of both CBT and diverse psychotherapeutic approaches, as delivered over an online modality (e.g., Dennis et al., 2020). Moreover, as brilliantly discussed by Swartz (2020), strategies for supporting patients during the COVID-19 pandemic can be found in virtually all psychotherapeutic disciplines. In this vein, therapists’ reluctance to use technology for psychotherapy may be related to uninformed attitudes, rather than fundamental issues relating to this modality (Van Daele et al., 2020). National and international institutions hold the responsibility for increasing the acceptance of telepsychotherapy among both clinicians and the general public, especially in the current context, given that a surge in the demand for mental health resources is expected in the months following isolation (Gao et al., 2020).

Surprisingly, we did not find any effect of psychotherapists’ clinical experience in predicting either the rate of interrupted treatments or therapists’ satisfaction with the online treatment modality. We hypothesize that, given the high correlation between psychotherapists’ age and clinical experience in our sample, this lack of evidence could represent a compensatory effect of the sample characteristics. Indeed, previous studies on psychotherapy treatments delivered *via* video-conferencing have shown that psychotherapists’ familiarity with Internet technology promotes patients’ compliance by limiting technical difficulties (e.g., brief interruptions or breakdowns in online communications; Etzelmueller et al., 2018) and that older age is associated with a lower dropout rate and better clinical outcomes (Catarino et al., 2018). In other words, younger therapists may encounter fewer technological barriers when delivering online sessions, whereas older therapists may benefit from their greater clinical experience, which allows them to better manage their patients during this potentially destabilizing transition in setting.

It is necessary to recognize the limitations of the present study. Notably, the enrolled psychotherapists performed their work mainly as private practitioners; thus, the generalizability of the present results should be limited to the changes in psychotherapy activities in the private practice. A further limitation relates to the cross-sectional design. Multiple measurement points in a longitudinal design would have the advantage of monitoring the provision of psychotherapy in Italy as the government restrictions eased. It should also be noted that the snowball technique used for recruitment may have produced a biased sample (e.g., the higher proportion of psychodynamic therapists may have been due to the therapeutic orientation of the authors). Finally, the study only analyzed psychotherapists’ self-reports, and no objective data (e.g., health insurance information) were considered.

To conclude, Table 1 reports key messages that can provide insight for countries struggling with the pandemic and offer specific guidance for policy makers, mental health institutions, professional organizations, and psychotherapists in promoting the continuity of psychotherapy treatment during the COVID-19 outbreak and in future pandemics.

**Table 1:** Key message for practitioners.

During the peak of the lockdown in Italy, 42.1% of psychotherapy treatments were interrupted. The following points are the factors we observed to limit the implementation of telepsychotherapy, followed by suggestions to help countries that are affected by the pandemic: **Therapists’ lack of experience with telepsychotherapy.** → Disseminate training programs on telepsychotherapy. **Patient’s lack of a private space to access telepsychotherapy sessions.** → Help and support patients to pragmatically access a private space. **Not using video-conferencing to administer telepsychotherapy (video-conferencing was the only remote modality found to predict therapists’ higher satisfaction).** → Therapists should use video-conferencing to deliver remote therapy, where possible. **Therapists’ consideration of teletherapy as compatible with their theoretical orientation (those who did not report less satisfaction).** → Relevant associations should seek to increase the acceptance of telepsychotherapy among both clinicians and the general public.

## Data Availability Statement

The raw data supporting the conclusions of this article will be made available by the authors, without undue reservation.

## Ethics Statement

The studies involving human participants were reviewed and approved by Ethics committee of the University of Padua. The patients/participants provided their written informed consent to participate in this study.

## Author Contributions

TB and AS developed the survey and wrote the first draft of the manuscript. AS analyzed the data. SS conceived the research study and contributed to the development of the survey. SS, VL, and FC contributed to the interpretation of the results and critically reviewed the final draft of the manuscript. All authors contributed to the article and approved the submitted version.

### Conflict of Interest

The authors declare that the research was conducted in the absence of any commercial or financial relationships that could be construed as a potential conflict of interest.

## Footnotes

^1^A growing body of literature found out the potential consequences of the COVID-19 pandemic scenario also in people with pre-existing mental health disorders (e.g., Moreno et al., 2020). For example, people with anxiety-related or mood disorders resulted in being significantly affected than others, reporting greater fear about the danger of contamination, socioeconomic consequences, xeno-phobia, and traumatic stress symptoms (Asmundson et al., 2020). Similarly, individuals at high clinical risk for psychosis may be prone to exacerbate psychological distress (DeLuca et al., 2020), as a result of heightened stress sensitivity and comorbid mental health problems (Boldrini et al., 2019).

^2^Therapists were asked to consider the all number of patients treated before the lockdown and to report (on different sliders, which could vary between 0 and 100%) the percentages of (i) interrupted treatments, (ii) treatments currently delivered by video call, (iii) by telephone, and (vi) face-to-face. Thus, all the distributions reported in *Changes in the Provision of Psychotherapy During the COVID-19 Lockdown* should be interpreted as the means and standard deviations of the percentages reported by enrolled therapists to the number of patients treated before the lockdown.

^3^The full model for the rate of interrupted treatments was as follows: rate ~ theoretical orientation × clinical experience × experience with online × theoretical compatibility × supervision + patient’s privacy (in Wilkinson notation).

^4^The *Δ*AIC was computed as the difference in AIC between the best ranked model and the null model, representing the difference in quality between the models.

^5^The full model for satisfaction was as follows: satisfaction ~ theoretical orientation + experience with online + theoretical compatibility + rate of interrupted treatments + dropped of supervision + telephone modality × clinical experience + video call modality × clinical experience + therapeutic modality (in Wilkinson notation).

^6^As pointed out by Racine et al. (2020), another therapeutic concern is the assessment of patients’ emotional states via telematic modality, especially about dissociation. Dissociative symptoms, which are challenging to assess even in natural therapeutic circumstances (e.g., Fagioli et al., 2015), could be more challenging to be identified using a video call, and barely impossible to identify via telephone.
