# Supplementary material for: Psychotherapy During COVID-19: How the Clinical Practice of Italian Psychotherapists Changed During the Pandemic
Source: Front Psychol. 2020 Oct 21;11:591170. doi: 10.3389/fpsyg.2020.591170 (PMC7641613; doi:10.3389/fpsyg.2020.591170)
Supplement: Supplementary file 1 [file Table_1.docx]

**Supplementary materials to:**

**Psychotherapy during COVID-19:**

**How the clinical practice of Italian psychotherapists changed during the pandemic**

**Tommaso Boldrini^1^, Arianna Schiano Lomoriello*^1,2^, Franco Del Corno^3^, Vittorio Lingiardi^4^, Silvia Salcuni^1^**

**1 Department of Developmental Psychology and Socialization, University of Padova, Padova, Italy,**

**2 Department of Cognitive System, Denmark Technical University (DTU), Copenhagen, Denmark.**

**3 Association for Research in Clinical Psychology (ARP), Milan, Italy.**

**4 Department of Dynamic and Clinic Psychology, Faculty of Medicine and Psychology, Sapienza University of Rome, Rome, Italy.**

***** **Correspondence:**

[arianna.schianolomoriello@unipd.it](mailto:arianna.schianolomoriello@unipd.it)

**Content:**

**Table S1: Summary of the dropout best model’s results.**

**Table S2: Summary of the satisfaction best model’s results.**

|  | **Participants’ rate of interrupted treatments** | | |
| --- | --- | --- | --- |
| *Predictors* | *Estimates* | *CI* | *p* |
| (Intercept) | 61.98 | 50.67 – 73.30 | **<0.001** |
| Previous experience in tele-psychotherapy [Rarely] | -11.53 | -18.36 – -4.70 | **0.001** |
| Previous experience in tele-psychotherapy [Frequently] | -28.46 | -38.41 – -18.51 | **<0.001** |
| Patient’s_privacy at home  [Yes] | 10.37 | 2.95 – 17.79 | **0.006** |
| Theoretical compatibility [Yes] | -6.29 | -14.44 – 1.86 | 0.130 |
| Theoretical orientation  [Others] | -5.82 | -16.28 – 4.63 | 0.274 |
| Theoretical orientation  [Psychodynamic] | -12.04 | -21.34 – -2.73 | **0.011** |
| Observations | 308 | | |
| R^2^ / R^2^ adjusted | 0.154 / 0.136 | | |

**Table S1. Summary of the dropout best model’s results.**

|  | **satisfaction** | | |
| --- | --- | --- | --- |
| *Predictors* | *Odds Ratios* | *CI* | *p* |
| (Intercept) | 0.53 | 0.19 – 1.37 | 0.193 |
| Participants’ rate of interrupted treatments | 0.99 | 0.98 – 1.00 | **0.012** |
| Previous experience in tele-psychotherapy [Rarely] | 0.50 | 0.28 – 0.90 | **0.022** |
| Previous experience in tele-psychotherapy [Frequently] | 11.47 | 2.22 – 210.78 | **0.020** |
| Theoretical compatibility [Yes] | 5.05 | 2.46 – 10.96 | **<0.001** |
| Videocall modality | 1.01 | 1.00 – 1.02 | **0.006** |
| Observations | 308 | | |
| R^2^ Tjur | 0.261 | | |

**Table S2. Summary of the satisfaction best model’s results.**
